# BioPS: System for screening and assessment of biofuel-production potential of cyanobacteria

**DOI:** 10.1371/journal.pone.0202002

**Published:** 2018-08-10

**Authors:** Olaa Motwalli, Magbubah Essack, Adil Salhi, John Hanks, Ivan Mijakovic, Vladimir B. Bajic

**Affiliations:** 1 King Abdullah University of Science and Technology (KAUST), Computational Bioscience Research Center (CBRC), Thuwal, Kingdom of Saudi Arabia; 2 Saudi Electronic University (SEU), College of Computing and Informatics, Madinah, Kingdom of Saudi Arabia; 3 Chalmers University of Technology, Division of Systems & Synthetic Biology, Department of Biology and Biological Engineering, Gothenburg, Sweden; 4 Novo Nordisk Foundation Center for Biosustainability, Technical University of Denmark, Lyngby, Denmark; Michigan State University, UNITED STATES

## Abstract

**Background:**

Cyanobacteria are one of the target groups of organisms explored for production of free fatty acids (FFAs) as biofuel precursors. Experimental evaluation of cyanobacterial potential for FFA production is costly and time consuming. Thus, computational approaches for comparing and ranking cyanobacterial strains for their potential to produce biofuel based on the characteristics of their predicted proteomes can be of great importance.

**Results:**

To enable such comparison and ranking, and to assist biotechnology developers and researchers in selecting strains more likely to be successfully engineered for the FFA production, we developed the Biofuel Producer Screen (BioPS) platform (http://www.cbrc.kaust.edu.sa/biops). BioPS relies on the estimation of the predicted proteome makeup of cyanobacterial strains to produce and secrete FFAs, based on the analysis of well-studied cyanobacterial strains with known FFA production profiles. The system links results back to various external repositories such as KEGG, UniProt and GOLD, making it easier for users to explore additional related information.

**Conclusion:**

To our knowledge, BioPS is the first tool that screens and evaluates cyanobacterial strains for their potential to produce and secrete FFAs based on strain’s predicted proteome characteristics, and rank strains based on that assessment. We believe that the availability of such a platform (comprising both a prediction tool and a repository of pre-evaluated stains) would be of interest to biofuel researchers. The BioPS system will be updated annually with information obtained from newly sequenced cyanobacterial genomes as they become available, as well as with new genes that impact FFA production or secretion.

## Introduction

Biofuels derived from cyanobacteria are recognized as a promising alternative energy resource [1, 2]. Consequently, select cyanobacterial strains have been engineered as cell factories for such a purpose [3–6]. Through genetic modifications aimed at disruption of competing pathways and overexpression of both endogenous and heterologous enzymes required for biofuel precursors, free fatty acid (FFA) producing cyanobacterial strains were engineered to increase the production and secretion of FFA. FFA associated research in this organism, as well as in *E*. *coli* and yeast, have primarily been focused on the naturally abundant long-chain (C14–C18) fatty acids (FAs) [7–11], even though medium-chain (C4–C12) fatty acid precursors were used to produce fuel with improved quality [12–14] and are known to be valuable industrial chemicals [15, 16]. These factors suggest that the increase in production and secretion of FFAs should be coupled with the tailoring of chain-length specificity.

In the period from 2010 to 2016, only three cyanobacterial strains have been experimentally evaluated and engineered for the FFA production. Experimental outcomes show that some strains were more efficient in producing biofuel than others [5, 17]. In 2010, Liu *et al*. [18] demonstrated enhanced FFA production through the engineering of the model organism *Synechocystis* PCC 6803, the first cyanobacterium to have its genome sequenced. Ruffing pursued the same path to enhance FFA production through engineering of two alternative strains: *Synechococcus* sp. PCC 7002, which has a faster growth rate [5], and *Synechococcus elongatus* PCC 7942, since its genome does not contain the polyhydroxybutyrate (PHB) pathway which competes for resources needed to produce FFA [6]. None of the strains engineered so far produced sufficient yields or titers of FFA to be considered economically viable for industrial-scale production [19].

To address this problem several *in silico* tools/algorithms have been developed, such as OptKnock [20], OptReg [21], OptGene [22], OptStrain [23], Ensemble Modeling approach [24] and the Genetic Design through Local Search (GDLS) [25], which aim at predicting engineering modifications for the optimization of targeted production. These tools collectively consider knockouts, overexpression and underexpression of genes, knock-ins of non-native functionalities, flux measurements from knockout and enzyme overexpression experiments of various reactions, and gene-protein-reaction (GPR) associations for achieving targeted overproduction. However, these approaches have several limitations that the OptForce tool [26] remedies to a certain extent by suggesting multiple engineering interventions for a wild-type strain that leads to metabolic flux data which forces a targeted overproduction. However, before these metabolic engineering strategies are used in the development of a microbial cell factory (MCF), a potential MCF chassis has to be selected. Currently, the evaluation of potential MCF is time-consuming and not efficient as it is done on a one-by-one basis. Thus, a more general approach that allows for all strains of interest to be screened simultaneously in order to identify putative chassis strains for targeted overproduction would be well received. This is exactly the niche that Biofuel Producer Screen (BioPS) covers, as it aims to select the good strain candidate, which then should be engineered using tools such as the ones mentioned above, that identify potential engineering interventions for the strain to optimize the production of the targeted product.

The criteria used for MCF selection are generally based on: 1/ identifying the MCF host requiring minimal metabolic perturbation as metabolic engineering usually compromises metabolic functioning (and the availability of genomic toolsets), 2/ availability of metabolic requirements (i.e., pathways, precursors, and cofactors) needed to ensure production and secretion of the product of interest, and 3/ toxicity of the product of interest and pathway intermediates. BioPS estimates the suitability of cyanobacteria based on their genetic characteristics expressed through their predicted proteomes, in order to single out potentially better biofuel producing chassis. Surprisingly, though more than 140 cyanobacterial sequenced genomes are available, they have not yet been systematically evaluated for their FFA production and secretion potential. Furthermore, the availability of cyanobacterial sequenced genomes allows for *in silico* screening of these strains for their potential to produce FFA [27], so that experimental evaluations can be focused only on the most promising strains. Based on the results from [27], we developed the BioPS screening system for cyanobacterial species, which estimates species potential to be successfully engineered for biofuel production. We believe that BioPS could help the selection of the most promising cyanobacteria strains with better potential for maximizing FFA production after suitable genetic engineering. BioPS is free for academic and non-profit use and can be accessed at (www.cbrc.kaust.edu.sa/biops).

## BioPS system: Design and implementation

BioPS is hosted on a CentOS 7.3 12 core virtual machine with 64 GB memory. The user interface is a typical HTML/Javascript front-end, backed by a PHP server scripting middle layer to handle front-end graphical user interface (GUI) and back-end (database and evaluation tool) functionality. BioPS back-end is comprised of two main components: 1/ the evaluation tool and 2/ the data repository. The evaluation process is an implementation of the adapted Free Fatty Acid Screen (FFASC) algorithm, an *in silico* screening method from [27]. Local installation of bioinformatic tools such as Basic Local Alignment Search Tool (BLAST) [28, 29], HMMER [30], and MATLAB [31] was used in this implementation. The process was optimized for high performance, including parallelizing BLAST processing by chunking the input genome into 100 data blocks (regardless of genome size), and scheduled to run 12 jobs concurrently using the GNU parallel scheduler (https://www.gnu.org/software/parallel/). The above set-up was optimal for the dedicated hardware resources. MySQL was used as our relational database system. BioPS was tested across major web-browsers such as Firefox, Chrome and Safari on Mac OS, Windows, and Linux platforms.

### Data sources

The list of 64 proteins that impact FFA production were retrieved from [27] and can be downloaded from the BioPS website with the associated information of relevance to FFA. In [27], it is reported that the list of 64 proteins was compiled based on a literature search for proteins relevant for FFA production demonstrated through genetic engineering of strains for FFA/biofuel production, as well as proteins required for fatty acid synthesis. For a detailed description of how Kyoto Encyclopedia of Genes and Genomes (KEGG) [32], Universal Protein Knowledgebase (UniProt) [33], and the Protein Families Database (Pfam) [34] were used for extraction of homologous proteins refer to [27]. From NCBI [35] we download 140 cyanobacterial genomes sequences and re-annotated them using the INtegrated Data Warehouse of MIcrobial GenOmes (INDIGO) pipeline [36], to ensure annotation consistency and standardized evaluation. Based on these re-annotated genomes, the corresponding predicted proteome sequences were derived.

### BioPS system structure

**Fig 1:**
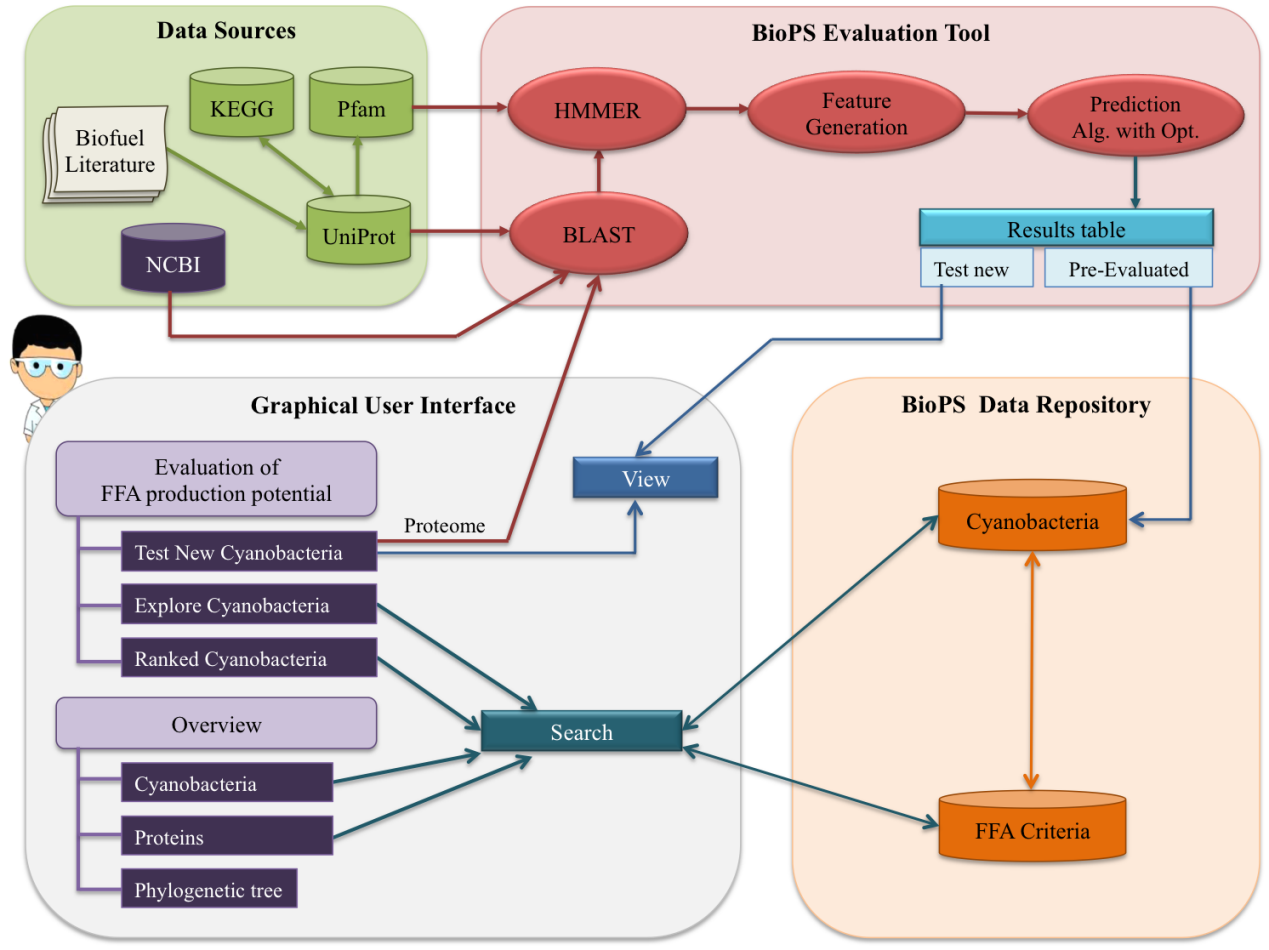
Global outline of the system platform and data flow: System structure overview. Arrow direction represents the flow of information between various modules of the system, where arrows (→) indicate the direction of flow of information, while bidirectional arrows (↔) indicate information flow in both directions, for example query sent to the module and results returned. Information used for the assessment (“FFA Criteria”), as well as pre-evaluated information for 140 cyanobacterial strains (“Cyanobacteria”), is saved locally in BioPS Data Repository and made available to end-users through the user interface.

BioPS system structure is comprised of four main components as shown in Fig 1, namely Data Sources, BioPS Evaluation Tool, BioPS Data Repository, and GUI.

### BioPS evaluation tool

The BioPS evaluation tool is an implementation of the established FFASC *in silico* screening method [27], which screens and estimates cyanobacterial strains for their potential to produce FFAs. This tool combines sequence homology (determined using BLAST) and domain search (determined using HMMER) to identify proteins to be considered in the evaluation, i.e. only homologous protein sequences containing all domains from the original protein are used for further analysis. The presence or absence of proteins in the list of orthologous groups is then used to generate the features to be processed by an optimized ranking algorithm that computes as a score the potential of the cyanobacterial strain to produce FFAs. Thus, the evaluation algorithm runs in two phases: 1/ The FFA feature generation phase, and 2/ the strain score computation phase, which produces a normalized score for the strain under evaluation, and based on this score, ranks the strain within the set of pre-evaluated cyanobacteria stored in the database. Depending on this rank the strain is assigned one of three categories: “Top-ranked”, “Positive” or “Negative” as a chassis FFA producer.

To be categorized as “Top-ranked”, strains are required to score higher than the positive reference strain *Synechococcus* PCC 7002. The “Positive” category includes strains that score lower than *Synechococcus* PCC 7002, but higher than *Pseudanabaena* sp. PCC 7367, while the rest of the strains will fall into the “Negative” category. The *Pseudanabaena* sp. PCC 7367 strain was used as a boundary between positive and negative strains, because its ranking position is the lowest for the set of strains that, based on K-means clustering, were placed in the same cluster as the positive reference strains.

### BioPS data repository

The main functionalities provided by the ‘Data Repository’ are the storage and retrieval of sequence and annotation data for proteins that either positively or negatively impact FFA biosynthesis, FFA production potential evaluation results, as well as general information for all pre-evaluated cyanobacterial strains. The list of proteins that impact FFA production and secretion will be extended through manual curation annually, as well as the list of cyanobacteria (where possible) and consequently the pre-evaluated cyanobacterial strain data.

### Pre-evaluated strains

The ‘BioPS Evaluation Tool’ was used to screen and rank 140 cyanobacterial strains with publicly available genome sequences. We retrieved all cyanobacteria genomes that are labelled as “Complete Genome” in the National Center for Biotechnology Information (NCBI) (http://www.ncbi.nlm.nih.gov/genome/browse/#). This is done to reduce bias in our ranking of strains due to the missing parts of genome sequences as our analysis is based on the presence and absence (hit number) of the 64 proteins we found relevant for the FFA production. Results for these are stored in the data repository along with information related to 64 relevant proteins. Out of the 140 pre-evaluated strains, 21 were categorized as “Top-ranked”, 49 as “Positive” and 70 as “Negative”.

Mapping protein sequences contained in the 140 cyanobacterial predicted proteomes against the protein sequences of the orthologous groups generated approximately 17,640 protein sequences. Of the 17,640 protein sequences, 2,678 were designated as proteins that positively impact FFA production, 10,856 as proteins that negatively impact FFA production, and 4,106 as proteins required for FFA production. These data have been stored in the ‘Data repository’ (see Fig 1).

### Graphical user interface (GUI) and utility

BioPS has a user-friendly web interface that allows for easy evaluation of new strains and analysis of pre-evaluated strains using the left-hand menu that includes three functionalities: “Test New Cyanobacteria”, “Explore Cyanobacteria” and “Ranked Cyanobacteria”. This left-hand menu additionally allows users access to cyanobacterial strain data housed in BioPS via the “Cyanobacteria”, “Proteins” and “Phylogenetic Tree” links.

#### Evaluation of FFA production potential

“Test New Cyanobacteria”. This link provides access to on-demand screening and evaluation of FFA potential for new cyanobacterial strains that are not included in the database. Testing a new strain can be accomplished by merely submitting its’ proteome sequence in FASTA format and providing the name for the submitted strain (see BioPS tool manual). The user will then receive the BioPS prediction results with the species score, evaluation category, strain recommendation, and the ranking position with respect to pre-evaluated strains.

Users are also provided “Analysis of strain results” which includes the orthologous groups hits present in the genome and the proteins with positive or negative impact on FFA production, or required for FFA biosynthesis. Based on these results, BioPS further provides “Suggested insertion/overexpression modifications to increase FFA production” and “Suggested deletion/under-expression modifications for proteins present in the organism with negative impact on FFA production”. Users can also visualize the present and absent FFA impact proteins for their strain through KEGG pathways (where the present proteins are highlighted in green and the missing proteins are highlighted in pink), listed under “Metabolic pathways” in the left-hand menu. Strain results are downloadable.

“Explore Cyanobacteria”. This link allows users to explore each of the 140 pre-evaluated cyanobacteria strains individually. After strain selection, evaluation and detailed information are displayed as for new evaluations, namely: (i) score, rank, and category, (ii) proteins impacting FA production for each strain, including sequence, annotation, orthologous, links to gene context in CyanoBase [37], as well as FFA impact details, and (iii) recommendations to increase FA production as suggestions for knockouts or gene inserts. This was done to avoid the re-evaluation of these strains on the fly, and thus provide fast data retrieval of evaluation results instead.

Here it should be noted that the proteins required for FA synthesis are classified into 12 orthologous groups (OGs). The well-studied model cyanobacteria such as *Synechocystis* sp. PCC 6803, *S*. *elongatus* PCC 7942, and *Synechococcus* sp. PCC 7002, contain all of the required OGs for FA synthesis. Additionally, out of the 140 evaluated cyanobacteria strains, 100 contained all 12 required OGs (inclusive of the model cyanobacteria). Meanwhile, 39 of the remaining cyanobacterial strains only contained 11 OGs. The protein that was not present in all these cases was EC:2.7.9.2 “pyruvate, water dikinase” (pps, K01007), which is involved in not only the pyruvate metabolism module but also in carbon fixation for cyanobacteria. Examples of such strains are *Candidatus Atelocyanobacterium thalassa* isolate ALOHA and *Prochlorococcus marinus* subsp. *pastoris* CCMP1986, where even the backup enzyme class that can catalyze the same metabolic step (“pyruvate, phosphate dikinase”, EC:2.7.9.1.) is also not present. This is as per our method which uses the re-annotated genomes from INDIGO and according to KEGG’s database. There is one case in which an additional required FA synthesis protein EC:2.3.1.12 “pyruvate dehydrogenase E2 component” (odhB, K00627) is absent. This protein EC:2.3.1.12 was considered not present in strain *Prochlorococcus marinus subsp*. *pastoris* CCMP1986, as a consequence of our stringent method which considers only the BLAST hits that have all protein domains of the query protein. Additionally, this case was mirrored with the protein EC:2.7.1.40 “pyruvate kinase” (pyk, K00873). So, only one of the evaluated cyanobacteria strains contained 10 OG’s (*Prochlorococcus marinus subsp*. *pastoris* CCMP1986), where the proteins EC:2.7.9.2 and EC:2.3.1.12 were not present. In conclusion, these cyanobacteria may be using alternative routes for synthesizing FA, that we do not know of. Nonetheless, the complete FA synthesis route is present for more than 70% of strains in BioPS.

“Ranked Cyanobacteria”. This link provides an aggregate view of the complete list of pre-evaluated cyanobacteria ranked based on species score. The “Top-ranked” strains represent candidate chassis strains that may be capable of producing and secreting FFA more efficiently than the currently engineered model strains. This view links each strain in the list to its associated “Explore Cyanobacteria” page as well.

When analyzing the BioPS ranked cyanobacteria (Table 1), we find positive reference strains *Synechococcus* sp. PCC 7002 (ranking position 22), *Synechocystis* sp. PCC 6803 (ranking position 24) and *S*. *elongatus* PCC 7942 (ranking position 39) outrank negative reference strains *A*. *platensis* NIES.39 (ranking position 114) and *Lyngbya* sp. PCC 8106 (ranking position 138) identical to results obtained in [27], with a slight change in raking positions due to 15 more strains being included in the analysis.

**Table 1:** Ranked list of cyanobacterial strains based on their FFA production potential score.

Ranking position	Ranked species	Values
1	*cyanobacterium endosymbiont of Epithemia turgida isolate* EtSB Lake Yunoko	1
2	*Prochlorococcus marinus* MIT 9211	0.9917
3	*Prochlorococcus marinus subsp*. *marinus* CCMP1375	0.9908
4	*Prochlorococcus marinus subsp*. *pastoris* CCMP1986	0.9786
5	*Prochlorococcus marinus* MIT 9301	0.9785
6	*Prochlorococcus marinus* MIT 9215	0.9768
7	*Candidatus Atelocyanobacterium thalassa isolate* ALOHA	0.9717
8	*Prochlorococcus marinus* NATL2A	0.9705
9	*Prochlorococcus marinus* NATL1A	0.9704
10	*Synechococcus* sp. CB0101	0.9702
11	*Synechococcus* sp. RS9917	0.9673
12	*Prochlorococcus marinus* MIT 9312	0.9667
13	*Prochlorococcus marinus* MIT 9202	0.9658
14	*Prochlorococcus marinus* MIT 9515	0.9652
15	*Thermosynechococcus elongatus* BP-1	0.9605
16	*Synechococcus* sp. WH 8109	0.9583
17	*Synechococcus* sp. WH 5701	0.9576
18	*Prochlorococcus marinus* AS9601	0.9569
19	*Thermosynechococcus* sp. NK55	0.9541
20	*Synechococcus* sp. JA-3-3Ab	0.9495
21	*Synechococcus* sp. CB0205	0.9486
22	*Synechococcus* sp. PCC 7002^+^	0.9433
24	*Synechocystis* sp. PCC 6803^**+**^	0.9303
39	*Synechococcus elongatus* PCC 7942^**+**^	0.8761
114	*Arthrospira platensis* NIES-39*	0.4286
138	*Lyngbya* PCC 8106 (CCY9616)*	0.0061

The list in Table 1 includes “Top-ranked” cyanobacterial strain that rank above S. PCC 7002 and the ranking position of all reference strains. Positive reference strains are marked with superscript + and negative reference strains with *.

**Fig 2:**
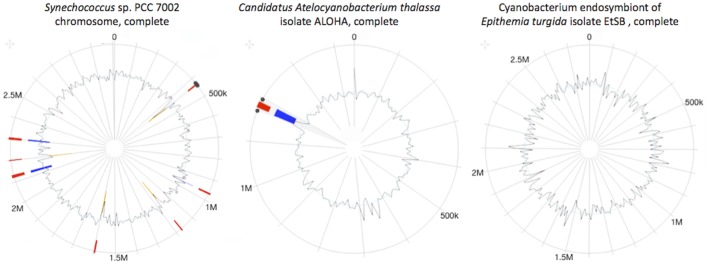
Genomic circular plots generated using Islandviewer 3. Islandviewer [44] depicts the prediction of genomic islands and virulence/resistance gene annotations using IslandPick (green), SIGI-HMM (orange), and IslandPath-DIMOB (blue).

Strains denoted as “Top-ranked”, ranked above *Synechococcus* sp. PCC 7002 at ranking position 22. Of these 21 “Top-ranked” strains, 20 were previously identified the “Top-ranked” in [27], thus only one new “Top-ranked” strain was added, namely “cyanobacterium endosymbiont of *Epithemia turgida* isolate EtSB Lake Yunoko” (ranking position 1). This *E*. *turgida* diatom endosymbiont is a nonphotosynthetic cyanobacterium [38]. The genome for this nonphotosynthetic cyanobacterium (EtSB) is reduced in size and consequently its gene set compared to known closely related strains. Specifically, it possesses the genes required for nitrogen fixation, but not for photosynthesis and thus it is dependent on its host cell [38]. This rare characteristic of cyanobacteria being nonphotosynthetic and having an evolutionary reduced genome makes this strain highly similar to the algal symbiont *Candidatus Atelocyanobacterium thalassa* (isolate ALOHA) (ranking position 7) [39]. Because streamlined genomes retain fewer foreign genes, we depict this potential similarity with respect to both strains having undergone some form of evolutionary metabolic streamlining, using the Islandviewer tool [40] to show that genomes of both strains have much fewer genomic islands than the reference strain *Synechococcus* sp. PCC 7002 (see Fig 2). This feature of these two strains is important as genome reduction experiments have demonstrated significant improvement in the yield of products of interest in *Escherichia coli* [41], *Bacillus Subtilis* [42] and *Corynebacterium glutamicum* [43]. Thus, it would be interesting to have an assessment of the FFA production potential of these strains. Additionally, the two cyanobacterial strains are symbionts of diatoms and algal strains, both of which have close relatives that show an increase in lipids when access to nitrogen is limited [44]. These observations suggest that the host and symbiont could be considered as an MCF unit to acquire higher yields of FFA, but the feasibility of such a combination has not been explored.

#### Browsing options

“Cyanobacteria”. Users can retrieve general information about stored cyanobacteria such as genome size, the total number of proteins and cell morphology. The user can view information for all strains or search for a specific strain by locating its genus, species, KEGG organism id, NCBI id, or CyanoBase id. Records are linked to KEGG, Cyanobase, NCBI BioProject [45], and the Genomes OnLine Database (GOLD) Project [46] whenever possible.

“Proteins”. FFA production impact proteins can be located using 4 different methods: 1/filtering by their impact type on FFA production: “Positive”, “Negative”, “Required”, or “All”, 2/searching by genes relevant to FFA production, where the user has the following options: “Insertion”, “Deletion”, “Overexpression”, “Underexpression”, “Present/Required”, and “All”, 3/searching by selected pathways such as “Fatty acid biosynthesis”, or 4/searching for information relevant to a specific gene using: Gene symbol, Locus tag, UniProt ID or KEGG orthology.

“Phylogenetic Tree”. Users can visualize the phylogenetic relationship among the pre-evaluated stored cyanobacteria through this view. The categories are color-coded: blue font denotes “Top-ranked” strains, green font denotes “Positive reference strains” and red denotes “Negative reference strains”.

#### Download

The user interface also provides a download option, where users are able to download in CSV format: 1/ FFA production impact proteins, 2/ Homologous proteins identified in all the predicted Cyanobacterial proteomes mapped to biofuel orthologous groups, and 3/ FFA production evaluation results for all cyanobacterial strains (http://www.cbrc.kuast.edu.sa/biops/download).

#### User manual

The user manual page (http://www.cbrc.kaust.edu.sa/biops/manual) provides more information on how to use the main functions of the system: A) Evaluation of FFA production potential and B) Browse Repository. In addition, the manual demonstrates how to interpret the results through the use of illustrative examples.

## Discussion and concluding remarks

The BioPS tool was created based on FFASC method, to allow researchers to easily screen and rank cyanobacterial strains for their ability to produce and secrete FFA based on the characteristics of their predicted proteomes. This is the first *in silico* tool developed for generalized screening of cyanobacterial strains for their potential as chassis FFA producers to focus experimental evaluations on the more promising cyanobacteria. The “Ranked Cyanobacteria” provides a snapshot of the successfully ranked pre-evaluated cyanobacterial strains, i.e. positive reference strains outrank negative reference strains. Additionally, BioPS show that “Top-ranked” strains are primarily unicellular and show phylogenetic closeness (see “Phylogenetic Tree”), all of which is identical to findings reported in [27].

In contrast to other *in silico* tools/algorithms (such as OptKnock, OptReg, OptGene, OptStrain, Ensemble Modeling approach, the GDLS algorithm, and OptForce) which suggest engineering interventions for targeted overproduction on the strain that is selected, BioPS performs a complementary task of suggesting the highly promising strains for successful engineering towards FFA production and secretion, and provides insight into how the strain of interest may perform relative to others. In this manner, BioPS fills in a new niche by addressing a need that was previously not covered, i.e. chassis selection for MCFs. Using BioPS for this purpose is less time consuming and is not dependent on the array of experimental data used in other non-*in silico* MCF chassis selection processes. Additionally, the BioPS repository component also differs from CyanoBase (cyanobacterial genomes and their functional annotation), CyanoClust (orthologs) [47], and cTFbase (transcription factors) [48], as these databases are specialized and not function-based, that is, BioPS is geared towards identifying cell factories for FFA production. Additionally, the proteins used in BioPS are manually curated. Like these tools and databases, BioPS has limitations as well, as the current version of BioPS uses the native biosynthetic capability for FFA production as the key consideration when identifying candidate cyanobacteria chassis strain, while there are other aspects such as environmental robustness, strain turnover rate, photosynthesis/CO_2_ fixating capabilities, gene expression levels, and metabolic flux, which we aim to add to the screening procedure in the future when more supporting data becomes available. Additionally, BioPS does not assess the predominant chain lengths for FFAs produced by the cyanobacterial strains. Thus, BioPS should serve as a complement to the existing tools and databases.

BioPS database will be expanded annually to include more strains and proteins that impact FFA production, and this screening of biofuel producing characteristics will be extended to other organisms in the future as well.
